# Establishing Reagent Testing Platforms for Functional Analyses in Sunflower

**DOI:** 10.3390/plants15010089

**Published:** 2025-12-27

**Authors:** Ryan A. Nasti, Cathy S. Kenderski, Aryaa Chanchani, Ambika Sharma, Benjamin K. Blackman

**Affiliations:** 1 Department of Plant & Microbial Biology, University of California, Berkeley, CA 94720, USA; nasti002@berkeley.edu (R.A.N.); ambikas1314@berkeley.edu (A.S.); 2 Department of Molecular & Cell Biology, University of California, Berkeley, CA 94720, USA; cathykenderski@berkeley.edu (C.S.K.); aryaac@berkeley.edu (A.C.); 3 Department of Integrative Biology, University of California, Berkeley, CA 94720, USA

**Keywords:** sunflower, *Helianthus annuus*, protoplast transfection, leaf infiltration, tissue co-culture, transformation

## Abstract

Recent advancements in molecular tools for plant genetic engineering, particularly CRISPR-based technologies, have created new opportunities for targeted genome editing. However, applying these tools remains challenging in crop species such as sunflower (*Helianthus annuus*) that lack established and effective transformation pipelines, including transient reagent delivery methods for functional screening and validation of genetic engineering tools. To address this gap, three major reagent delivery platforms, namely protoplast transfection, leaf infiltration, and *Agrobacterium*-mediated tissue co-culture, were systematically adapted and assessed for use in sunflower seedlings. While each method enabled successful reagent delivery, they differed in their levels of scalability and efficiency. With these platforms, delivery by different *Agrobacterium* strains and the effectiveness of various reporter gene expression cassettes were compared to define the most experimentally suitable components for different applications in sunflowers. Together, these results establish a foundational toolkit for transient functional testing in sunflower and pave the way for more sophisticated genetic engineering approaches in this agriculturally important oilseed, confectionary seed, and horticultural crop.

## 1. Introduction

The molecular toolkit for plant genetic engineering has been subject to major advancements and expansions over the last two decades. Traditionally, genetic manipulation has involved integrated transgene cassettes or random mutagenesis [1]. Recent advancements, such as the advent of CRISPR-based gene editing, have greatly expanded the possibilities for genetic manipulation [1,2,3]. The variants introduced by these tools have become highly sophisticated, growing from the non-specific indels primarily created by the original Cas9 editing reagents [3] to the more precise single-base changes and direct sequence inserts that can be introduced with base editors and prime editors, respectively [4]. These technological advancements open the door to many new types of engineering but come with several remaining challenges.

One fundamental remaining challenge is that new genome engineering reagents from promoter sequences to guide RNAs (gRNAs) to reporter genes vary in their functionality across species or even across genomic targets [5,6,7]. Consequently, to properly and effectively use these tools, having a suite of methods to screen and optimize reagent functionality before embarking on more extensive engineering efforts can be useful, especially if making transgenic lines in a given species requires a substantial investment of time, effort, and funding. One often applied form of functionality test is transient reagent delivery to tissues of interest [8,9]. In certain species, there are many available options for reagent testing, whereas in other species have few or no well-developed methods. For instance, a rich toolkit of reagent testing methods has been developed in *Nicotiana benthamiana* [10]. In particular, leaf infiltrations have consistently been used for reagent testing in *Nicotiana* sp. [11], and have been adopted in select other species [12,13]. Beyond testing molecular components, leaf infiltrations have additional useful applications, including the delivery of non-transgene materials such as nanoscale biosensors [14]. Yet, when compared to other methods, leaf infiltrations can prove challenging to implement at scale, as transfecting a suitable amount of tissue without compromising the assay by inflicting too much leaf damage can be difficult in many species [11].

Protoplast transfections are one good alternative to leaf infiltrations and have become a highly used reagent testing platform. This approach has been applied across a wide range of plant species from monocots like rice and maize to eudicots like tobacco and tomato [15]. One key advantage is the scalability of protoplast isolations, since the millions of harvested cells can be subsequently split across different experimental treatments in 48- or 96-well plates. Additionally, because the cells are stripped of their cell walls during the process of protoplast isolation, they are easier to image and thus have many useful applications, including cell sorting [16] and subcellular investigation of protein function [17]. Although this approach’s scalable nature allows for testing of many parameters in each experiment, the process of isolating and transfecting protoplasts remains tedious and technically challenging, and in need of troubleshooting in many systems, in part due to their fragility with overly rigorous handling [18].

While leaf infiltrations and protoplast transfections have opposing strengths, ease and high transfection rates versus high scalability, respectively, these two alternatives do not cover the full space of possible reagent delivery scenarios. For many situations, an intermediate method with modest scalability but still efficient delivery would be of great benefit. One such method that fits this niche is the co-culturing of seedlings or explants in an *Agrobacterium tumefaciens* solution. Co-culture techniques involving whole seedlings or explants rely on growth in sterile culture, then transfer into an *Agrobacterium* solution treated to increase the bacteria’s virulence [19,20,21]. After the co-culture period, relatively high rates of gene transfer are observed in the tissues exposed to the *Agrobacterium*. Such methods have been employed in seedlings across many species, including both monocots and dicots [19,21].

Although extensive efforts have established reliable transient reagent testing platforms in model plant systems, reagent delivery methods remain underdeveloped for many crops and non-model species. One crop in need of further methods development is the oilseed, confectionery seed, and horticultural crop sunflower (*Helianthus annuus*). Ample research has described and mapped the genetic basis of variation in many traits relevant for fundamental biological or agricultural problems, but at present, there is very limited capacity to test the functions of associated candidate genes while working in sunflower itself [22,23,24,25]. This limitation is partly due to the shortage of established and reliable reagent delivery and germline transformation platforms for sunflower [26]. In sunflowers, some useful methods for transient reagent delivery to sunflower tissues have been advanced, most notably the successful use of vacuum infiltration to transfect leaf disks [27], but there are many opportunities to extend this toolkit. Therefore, looking to expand reagent testing capabilities for sunflower to support future genome engineering applications, we adapted, performed, and evaluated the efficacy of each of the commonly employed reagent testing methods—leaf infiltrations, protoplast transfection, and *Agrobacterium* co-cultures—in sunflower seedlings.

## 2. Results

### 2.1. Protoplast Transfections Enable Modest Reagent Delivery Efficiencies

**Figure 1:**
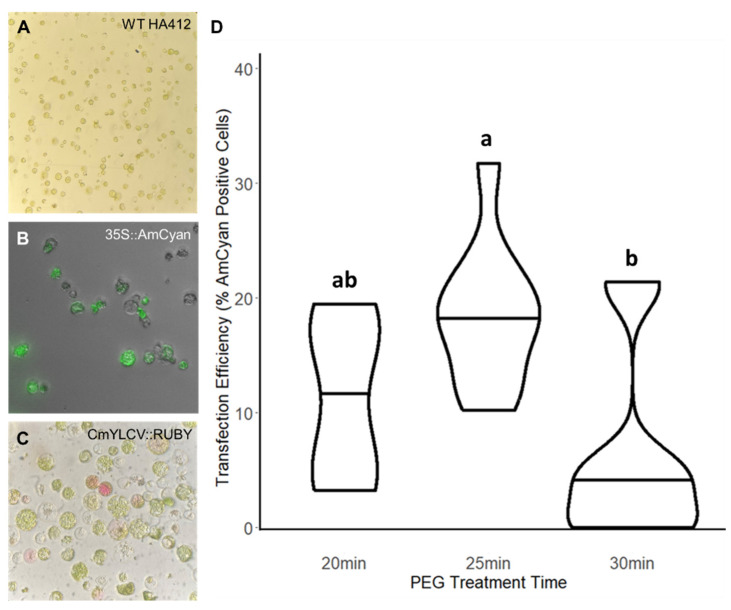
Optimizing Protoplast Transfections. Protoplasts are isolated from the first emerging true leaves of sterilely grown sunflower seedlings. Once these digested protoplasts are isolated (**A**), they can be transfected with plasmids including the fluorescent reporter, 35S::AmCyan (**B**), or the pigment-producing CmYLCV::RUBY reporter (**C**). To ensure a high transfection rate, the treatment time with PEG was tested in 5 min increments (**D**). The 25 min PEG treatment was found to be most effective, exceeding a 30% transfection rate in some trials. Different letters indicate significant differences in the mean between treatments based on post hoc tests.

We developed a protoplast isolation method by adapting a previously used *Arabidopsis thaliana* method [28] to isolate sunflower protoplasts, including important adjustments to obtain high numbers of protoplasts with minimal debris (Figure 1A). Most notably, the source of the starting material impacts the amount of extra debris present amongst the isolated cells. If the cotyledons are included in the starting plant material, the amount of debris is much higher compared to using only the first true leaves (Supplemental Figure S1). Avoiding the cotyledons and isolating protoplasts from the first true leaves, roughly one million cells can be collected from the leaves of ~8 sunflower seedlings.

We then proceeded to optimize reagent delivery for isolated protoplast cells. We tested two reporters for positive transfection of the isolated protoplasts: the fluorescent protein AmCyan [29] and the gene cassette that produces the pigment RUBY [30]. When assessing the level of reporter signal in transfected protoplasts, we observed that plasmid input concentration was directly correlated with observable signal. We only observed transfection rates higher than 10% when a plasmid input greater than 15 ug (at a concentration of approximately 250 ng/uL) was used (Supplemental Figure S1G). At this plasmid input concentration, reporter signal was evident using either AmCyan (Figure 1B) or RUBY (Figure 1C), but limited to approximately 15% transfection efficiency. When the plasmid input was increased beyond 15 ug to 17.5 ug, transfection efficiency remained at similar levels albeit with decreased variability across the trials (Supplemental Figure S1G).

Looking to improve these transfection rates, we adjusted the polyethylene glycol (PEG) incubation time to test the impact on transfection. PEG is a challenging parameter to modulate, as excessive exposure to PEG can cause more cells to burst, thus negatively impacting transfection efficiency (Supplemental Figure S2). To more subtly alter PEG exposure, we opted to extend PEG treatment time instead of adjusting PEG concentration. We tested three PEG treatment times differing by 5 min increments (20, 25, and 30 min), and we observed a considerable increase in transfection efficiency with extended exposure, peaking at 30% for a 25 min exposure (Figure 1D, Supplemental Figure S2H). While this efficiency is lower than what has been achieved in certain highly optimized systems like *Arabidopsis thaliana* or rice (62% and 80%, respectively) [31], this efficiency is within the range achieved in less efficient but successfully implemented protocols in other species [32]. At a PEG treatment time of 30 min, individual replicates showed similarly high transfection rates, but those increases were offset by an increase in cell death in other replicates (Supplemental Figure S2C,D,H). Consequently, we observed greater variability across trials for the 30 min PEG treatment (Supplemental Figure S2H), indicating that a 25 min PEG treatment is optimal. Altogether, these experiments established a functional protoplast transfection system in sunflower for reagent testing, albeit with the potential to be optimized further increases in efficiency.

### 2.2. Leaf Infiltrations Provide High Localized Reagent Delivery

**Figure 2:**
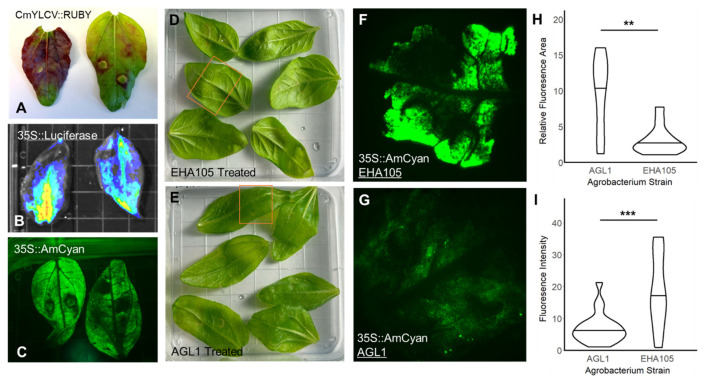
Applying Leaf Infiltrations for Reporter and *Agrobacterium tumefaciens* Strain Testing. Using the first emerging true leaves from sterilely grown sunflower seedlings, *Agrobacterium* strains encoding individual reporter gene cassettes were perfused into the leaves: CmYLCV::RUBY (**A**), 35S::Firefly Luciferase (**B**), 35S::AmCyan (**C**). The more mature the leaves, the more ‘patchy’ the delivery was ((**A**), **right**) compared to the younger leaves ((**A**), **left**). With this leaf infiltration platform, two *Agrobacterium* strains, EHA105 and AGL1, were selected for comparison to determine their relative transfection rates. By imaging the area of perfusion in infiltrated leaves ((**D**,**E**), orange box), the total area and intensity of AmCyan transgene cassette delivery were determined (**F**–**I**). The two strains showed different delivery tropisms, with EHA105 delivering more intensely to a smaller area ((**F**,**H,I**); relative fluorescence area = area within the imaged section with fluorescent signal present, fluorescence intensity = pixel intensity within the fluorescent area) compared to the more diffuse but broader delivery area seen with AGL1 (**G**–**I**). ** *p* < 0.01, *** *p* < 0.001.

Since the observed protoplast transfection rate (<30%) was lower than what is often needed for an observable signal for many reagent types, we turned to leaf infiltrations. Though less scalable than protoplast transfection, reagents are usually delivered to a greater fraction of the treated tissue. Leaf infiltrations are most utilized in *Nicotiana* species because they have highly permissive features that lead to greater uptake of *Agrobacterium* through syringe perfusions or vacuum infiltration and delivery to most, if not all, of the leaf area with a single perfusion [11]. In contrast, other species tend to be more challenging [11,33], with smaller total delivery areas per single syringe perfusion that are often limited to the leaf area near the perfusion site. We observed that syringe perfusions often resulted only in delivery local to the perfusion site (Figure 2A, right, Supplemental Figure S5A,B). Reasoning that younger leaves may possess more amenable stomata than less permissive older leaves, we tested the impact of leaf developmental stage. Consistent with our hypothesis, we were more reliably able to deliver *Agrobacterium* solution to a greater area by syringe perfusion in younger leaves (Figure 2A, left). Using these leaf infiltration parameters, we successfully delivered RUBY (Figure 2A), AmCyan (Figure 2B), and luciferase (Figure 2C) reporters to most of the area of perfused young leaves.

### 2.3. Comparing T-DNA Transfer Capabilities of Different Agrobacterium Strains with Leaf Infiltrations

*Agrobacterium tumefaciens* is widely used across many plant species for the transfer of gene cassettes into plant tissues [34,35]. However, different *Agrobacterium* strains vary in how well they infect and transfer genetic material in different species and even tissues within species [35,36,37]. Therefore, we conducted a set of leaf infiltration experiments comparing two *Agrobacterium* strains to determine which was most useful for this application in sunflower leaves. We chose specifically to compare the strains EHA105 and AGL-1. EHA105 is a commonly used strain in sunflower experiments, having been successfully employed in seedling vacuum infiltration experiments [38]. AGL-1, on the other hand, has been less extensively used, but where it has been tested in sunflowers, it has performed better than another more commonly used strain, LBA4404 [39]. With these two strains, we can therefore compare a promising, albeit limited in use to date, strain against one of the standards for the system.

To compare the reagent delivery by EHA105 and AGL-1, we transformed each strain with the 35S::AmCyan reporter and performed perfusions into young sunflower leaves. Interestingly, the two strains had inverse results in terms of signal and delivery area. Using EHA105 for delivery yielded a patchier delivery area but greater reporter signal in transiently transformed sites (Figure 2D,F,H,I). In contrast, AGL-1 delivery was characterized by a more consistent fluorescence signal across the delivery area with fewer highly expressing puncta (Figure 2E,G–I). These contrasting results present these two strains, then, as a pair of complementary tools. Use of EHA105 may be more effective for applications where a greater amount of reagent is needed locally. In contrast, AGL-1 may be more effective for applications where a weaker, more diffuse, but widespread reagent delivery pattern is desired, as may be the case when reagents that affect signaling through developmental or hormonal pathways have dosage-dependent impacts, or when reagents are toxic to cells if expressed at high but not low doses.

### 2.4. Relative Promoter Element Expression Testing with Dual Luciferase Assays

**Figure 3:**
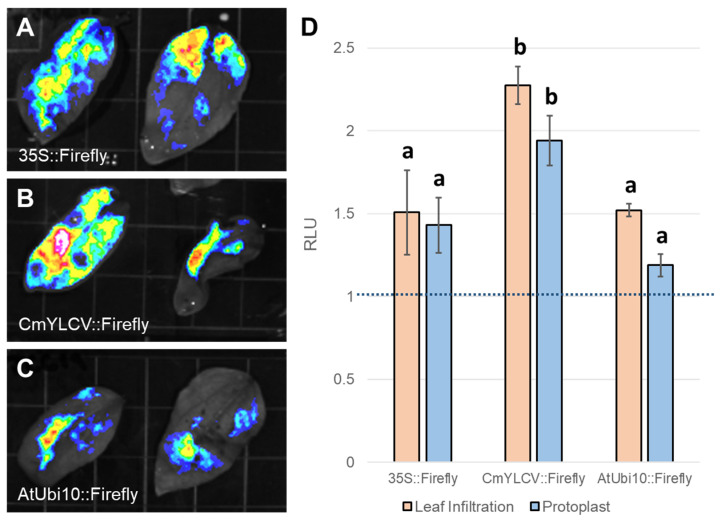
Using the Dual-Luciferase Assay to Quantitatively Compare Promoter Expression Levels. Looking to define the most effective promoters for constitutive gene expression in sunflower tissues, three promoters were selected to test in sunflower leaf infiltrations: 35S (**A**), CmYLCV (**B**), AtUbi10 (**C**). Using these promoters to drive firefly luciferase, *Agrobacterium tumefaciens* strains encoding these gene cassettes were infiltrated into sunflower leaves for qualitative assessment (**A**–**C**). Using these same firefly luciferase gene cassettes to compare to nos::Renilla luciferase with the Dual-Luciferase assay, it was found that the CmYLCV promotes the highest gene expression after both leaf infiltration and protoplast transfection compared to either the 35S or AtUbi10 promoters (**D**). Within methods, different letters indicate significant differences in mean expression between promoters based on post hoc tests.

Expression controlling elements, like promoters and terminators, are important components of genetically coded molecular reagents and also require optimization for species, tissue, and application [40]. For instance, the subset of promoter sequences most commonly used across species for high constitutive expression levels can vary considerably in their exact expression patterns. In particular, despite often driving high levels of constitutive expression in other species like *A. thaliana*, the 35S promoter has been observed to yield less substantial expression levels in sunflower and other species in Asteraceae [41,42]. We chose to compare the expression level of 35S (Figure 3A) to two other promoters often used for high constitutive expression, AtUBQ10 (Figure 3B) and CmYLCV (Figure 3C), thus determining their utility to drive reagent expression in sunflower. To do so quantitatively, we conducted dual luciferase reporter assays, a first use of this method in sunflowers. The *Agrobacterium* cultures used for this reagent testing approach each carry two gene cassettes: one in which the promoter of interest drives Firefly luciferase expression and another in which the nos promoter drives expression of Renilla luciferase. Since these cassettes are self-normalized, results from different experiments with varied transfection rates can be compared. Consequently, we were able to test the effectiveness of each promoter in both leaf infiltrations and protoplast transfections to see if the relative expression levels driven by the three promoters are consistent across different target tissues. Notably, across both delivery approaches, the relative expression levels for the three promoters did remain consistent, with CmYLCV driving the highest expression and 35S and AtUBQ10 being rather similar, although the specific magnitude appears slightly lower in the protoplasts than in leaf infiltrations (Figure 3D). Based on these results, the CmYLCV promoter appears to be the best choice for applications seeking to drive the highest level of expression, but all three promoters are functional in multiple sunflower tissues.

### 2.5. Applying Delivery Methods to Determine Gene Editing Reagent Efficacy

**Figure 4:**
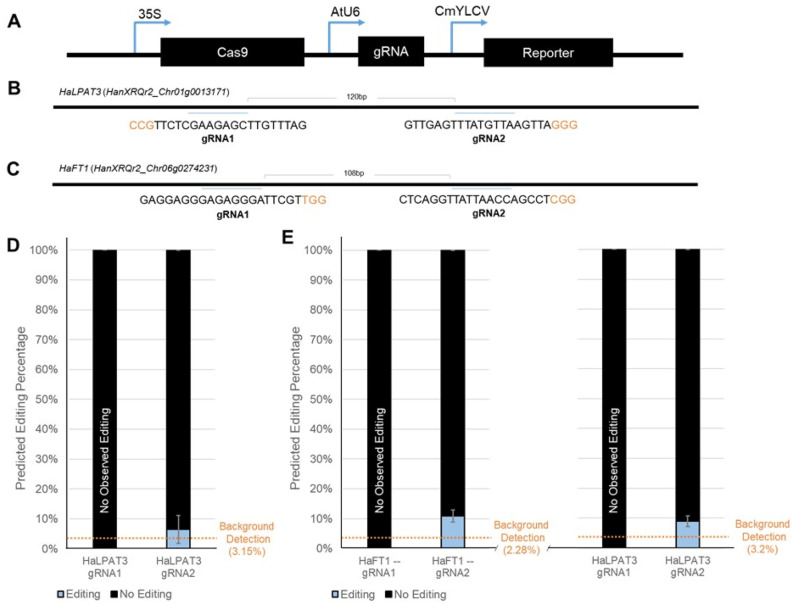
Low Gene Editing Rates Observed. The delivered constructs constitutively expressed Cas9, a gRNA cassette, and a robustly expressed reporter gene, either RUBY or AmCyan (**A**). For each of the two targeted genes, *HaFT1* (HanXRQr2_Chr06g0274231) (**B**) and *HaLPAT3* (HanXRQr2_Chr01g0013171) (**C**), two unique gRNAs were designed to target different portions of the coding sequence (PAM sites shown in orange font). As quantified by TIDE, the gene editing rate achieved in RUBY-positive protoplasts was low and not distinct from background detection (**D**). Gene editing rates achieved by leaf infiltrations with strong reporter signal (**E**) were also low but above background prediction levels compared to non-editing control traces in two cases.

With serviceable reagent delivery efficiency in place, we performed initial proof-of-concept experiments to determine whether the effectiveness of CRISPR reagents (Figure 4A), specifically the efficacy of individual gRNAs, could be tested in vivo with either protoplast transfection or leaf infiltration. We designed gRNAs for two targets, *HaFT1* and *HaLPAT3* (Figure 4B,C); the editing efficiencies of two guides for each target were examined. After successful transfection of protoplast cells with a 35S::Cas9/AtU6::gRNA cassette, as verified visually by RUBY reporter expression, genomic DNA was extracted and treated with an off-target restriction enzyme to enable greater PCR amplification (Supplemental Figure S3). Then, the target region was amplified by PCR. After reporter screening to confirm successful reagent delivery, genomic DNA was isolated and amplified from the protoplast cells (Supplemental Figure S4). Despite the guides passing design parameters, when tested in protoplasts, only very low levels of editing indistinguishable from background (~6.7% ± 4.7) were observed (Figure 4D).

We hypothesized that the relatively low transfection rates for reagent delivery in the protoplasts may explain these observations of inefficient gene editing. Therefore, we pivoted to test the guides with leaf infiltrations. Once again, we extracted DNA from RUBY reporter-expressing tissues (Supplemental Figure S5) to amplify and assess gene editing efficiency. Despite observable reporter expression in the tissues collected, similarly low editing efficiencies were observed (*HaFT1* gRNA2 10.83% ± 1.98, *HaLPAT3* gRNA1 9% ± 1.85) as compared to the protoplast transfections (Figure 4E). These initial results may indicate either that the efficiency across all four guides is low or, more likely, that one of the gene editing reagent components could be expressed more effectively. Different components of the gene editing cassette, like Cas9, could be adapted to utilize higher-expressing promoters like CmYLCV instead of 35S. Additionally, tissue-specific or inducible promoters could be employed to selectively express gene editing reagents in a more controlled manner. The delivery techniques we have developed can be amended to assess such interventions and, upon establishment of a fully effective screening pipeline, be applied in the optimization of gene editing reagents for successful use in generating targeted lesions in the future.

### 2.6. Intermediate Delivery Capabilities Observed with Agrobacterium and Cotyledon Co-Cultures

**Figure 5:**
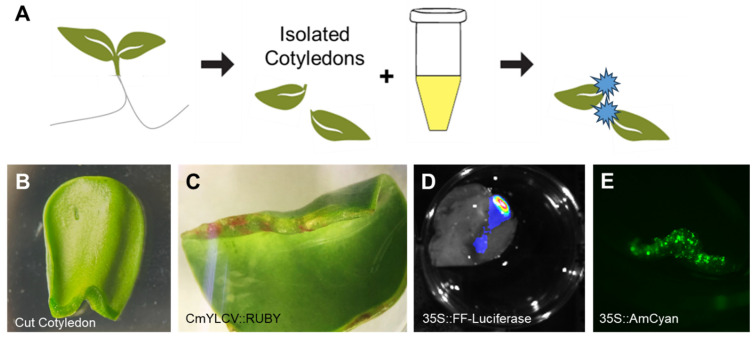
Establishing Cut Sunflower Cotyledon and *Agrobacterium tumefaciens* Co-Culture. From seedlings germinated in liquid ½ MS solution, cotyledons are cut at their base before co-culturing in a treated *Agrobacterium* solution from which reporter delivery is observed (**A**). The delivery localized at the cut site on the isolated cotyledons (**B**), which was demonstrated with multiple reporter cassettes: CmYLCV::RUBY (**C**), 35S::Firefly luciferase (**D**), 35S::AmCyan (**E**).

Looking to establish a more intermediate method to more effectively combine scalability and transfection efficiency, we turned to seedling and *Agrobacterium* co-culture. Such methods have been used with high efficacy in species like *A. thaliana, N. benthamiana*, and other solanaceous crops [19,20]. Despite the success in those systems, such methods have proved more challenging in seedlings with a waxier layer around their cotyledons, as is the case in sunflower seedlings. To overcome this barrier, we cut the cotyledons off young seedlings before culturing with the treated *Agrobacterium* (Figure 5A). This action creates a wound site that enables improved exposure to *Agrobacterium* infection (Figure 5B). At this wound site after *Agrobacterium* co-culture, we observed reliable reporter gene signal for three reporters: CmYLCV::RUBY (Figure 5C), 35S::Luciferase (Figure 5D) and 35S::AmCyan (Figure 5E). While this delivery is very localized, the tissue area is considerable, and cotyledons can be individually cultured in multi-well plates. While unable to reach scalability at the order of protoplasts, which can have individual treatments spread across 48- or 96-well plates, cotyledon co-cultures are generally constrained to experiments using 6- to 24-well plates, depending on the size of the seedlings or explants used. The ability to deliver transgenes by co-culture to explant tissues that can then be cultured in plates, therefore, still allows for a level of scalability that is not achievable with infiltrations, which require even greater growth space per experimental replicate. For example, a tub of 10 sterilely grown sunflowers will have 20 initial true leaves for infiltration. In the same space, one could have multi-well plates containing as many as 96 treated cotyledons, and even more if the plates are stacked. Thus, this intermediate experimental scale enables greater throughput and maintains tissue-level reagent delivery.

## 3. Discussion

Pipelines for genome engineering are more effective when supported by well-developed methods for reagent testing. However, these methods are not robust or comprehensive in many systems, including our focal study system, sunflower. Therefore, we sought to address this gap by testing the performance of several widely used reagent delivery techniques—protoplast transfections, leaf infiltrations, *Agrobacterium* and tissue co-cultures—in sunflower seedlings. By varying important parameters, we demonstrated replicable and effective protocols for each technique, as validated with multiple reporter cassettes. Importantly, we performed reagent-specific optimization for several factors, enabling us to identify optimal input levels and PEG treatment times for protoplast transfection (Figure 1), high-expression promoters for leaf infiltrations (Figure 3), and *Agrobacterium* strains with higher transfection rates (Figure 2). The ability to test and isolate different reagent components with these methods will allow for direct optimization of gene cassettes that can then be applied for plant genome engineering and crop improvement efforts.

Having a versatile methodological toolkit enables greater strides to be made towards plant engineering and biotechnology. Even though each of these methods has a mix of strengths and drawbacks, their unique features suit them to addressing distinct technical challenges. These unique features are most directly related to the ratio of scalability and overall reagent delivery to a given tissue. In the case of leaf infiltrations, the high proportion of transfected tissue makes them very useful in applications like the production of metabolites or proteins. They are also useful for assessing tissue-level processes like cell-to-cell mobility, which can be assessed through co-infiltration of transcriptional and translational reporter gene cassettes, or hypersensitive responses to pathogen effectors [43,44,45,46]. Leaf infiltrations also have been widely employed in species like *N. benthamiana* to produce compounds such as antibodies and vaccines [47] or other natural products [48].

Conversely, protoplast transfections are more favorable for large-scale experiments where many factors can be examined simultaneously. In sunflowers, there is a substantial need to further validate additional components to expand the applicable molecular toolkit. We only tested a small number of promoters that are currently widely used in other species (Figure 3). We have demonstrated that the dual luciferase platform can be successfully employed in sunflowers, and this method may be used to test additional candidate promoter sequences for expression level or tissue specificity at scale. For instance, non-coding sequences adjacent to genes expressed in desirable tissues or environments, as determined from the sunflower expression atlas database SunExpress (https://www.heliagene.org/sunexpress.html) or equivalent databases for other Asteraceae crops (e.g., https://lettuce.bioinformatics.nl/LEB/), could be validated for promoter activity using this method. Moreover, this method may be adapted to compare expression from allelic promoter sequences for defining *cis*-regulatory elements that harbor variation relevant to evolutionary or agronomic traits of interest, or to test the impacts of proposed genome edits to regulatory sequences on expression [49]. Extending beyond promoters, we found that CRISPR/Cas9 gene editing reagents may require substantial species-specific optimization for use in sunflower, as we observed low levels of editing for only a subset of the experimental reagents we tested. Using protoplasts to screen additional promoters, Cas9 variants, and gRNA targets should allow identification of the most effective combination(s) of tools to enable gene editing in sunflower tissues.

Tissue co-cultures with *Agrobacterium* have the advantage of scalability like protoplasts, but have the added benefit of involving whole plant tissues. This approach can be leveraged going forward to establish additional reagent testing platforms in sunflower. For example, two applications of tissue co-culture could be to test tissue-specific binding of a transcription factor to a regulatory sequence normalized with the dual luciferase assay [50,51] or delivering developmental gene overexpression cassettes to potentially overcome regeneration bottlenecks [52]. Expanding these co-culture techniques beyond cotyledon explants can provide a more rapid assessment platform to examine such reagents in the tissues where the focal genes are natively expressed [53]. Further improvements may also be made to each of the methods themselves for increased tissue transfection. Some potential opportunities for such improvement could include alteration of PEG or enzyme concentrations in protoplast transfections or the inclusion of mild wounding through sonication [21] as part of the co-culture method.

Some recent interesting progress has been made for reagent testing in sunflower and other challenging crops. One example is the use of robotics with nanomaterials, but these methods can carry high upfront equipment costs that limit their accessibility [54]. Here, by establishing several broadly accessible techniques in sunflowers, we have expanded the biotechnology toolbox towards leveraging the extensive knowledge gained from past genetic and genomic studies of this crop and its wild relatives for future fundamental science or applied engineering applications. To fully realize this potential, many existing or new molecular reagents will need to be functionally evaluated for use in sunflowers. Building reagents using components highlighted in this study (promoters: CmYLCV, AtUBQ10; reporter genes: Firefly luciferase, RUBY, AmCyan) and testing them with the methods outlined will enable their functional validation before use in plant-scale engineering efforts. As a system, sunflower still has major barriers with tissue regeneration and thus genetic transformation [26]. Nonetheless, the reagent delivery methods we have evaluated mark considerable steps towards advancing sunflower biotechnology, particularly in the case of co-culture, which can deliver tools that can reduce or remove some of the downstream transformation barriers in sunflower tissues. In addition, the series of experiments we have conducted provides a framework for how such assays could be developed in other horticultural and ornamental crops currently lacking such methods.

## 4. Materials and Methods

### 4.1. Plant Material

Seeds from the cultivated sunflower accession HA412 HO (USDA GRIN ID: PI 642777) were used for all reagent testing experiments, as they germinate quickly and uniformly at approximately seven days.

### 4.2. DNA Construct Assembly

The majority of DNA constructs were assembled using a published Golden Gate cloning toolkit and the related methodologies, including a suite of promoters and T-DNA backbone vectors (Supplemental Table S1) [55]. Additional preassembled plasmids, previously used to test promoter expression strength, were obtained from the Voytas lab (Supplemental Table S1, External Plasmids) [56].

### 4.3. Protoplast Isolation and Transfection

Protoplasts were isolated from true leaves of sterilely grown sunflower seedlings. The seeds were dehulled, sterilized in a 50% bleach solution for 5 min, and washed three times with sterile ddH20 before plating into 16 oz Pro-Kal deli-style tubs (Fabri-Kal, Kalamazoo, MI, USA) containing 1/2 MS media with 0.5% sucrose (*w*/*v*). Plants reach the appropriate stage for tissue collection roughly 3–5 weeks after plating (~3 weeks and ~5 weeks when grown in 21 °C and 15 °C, respectively, in 10 h light: 14 h dark photoperiod conditions), or when the first true leaves have established. We processed seedling leaf tissue by chopping it first into strips and then into smaller cut bits with a razor blade. We observed that protoplasts isolated from just the true leaf tissue were cleaner and had less additional debris than protoplasts isolated from both the cotyledons and true leaves (Supplemental Figure S1A–F).

Cells were incubated overnight (12–18 h) in 15 mL of enzyme solution [Cellulase R10 (1.5% *w*/*v*), Macerozyme R10 (0.4% *w*/*v*), 20 mM MES, 0.4 M Mannitol, 20 mM KCl, 10 mM CaCl_2_, BSA (0.1% *w*/*v*)] to break down the cell walls, while shaking at 15 rpm. After priming with 2 mL of buffer W5 (2 mM MES, 154 mM NaCl, 125 mM CaCl_2_, 5 mM KCl), we used a 40 µM Falcon nylon filter (Corning, Tewksbury, MA, USA) in a sterile Petri dish to slowly filter the protoplast solution transferred with a 25 mL serological pipette. The filtered protoplast solution was transferred to two separate 50 mL Falcon tubes, each containing 15 mL 0.55 M sucrose solution. The tubes were centrifuged at 1000× *g* for 5 min at room temperature, and in order to fractionate in the sucrose gradient, deceleration was turned off on the centrifuge (~25 min for the centrifuge to stop completely). Using a 25 mL serological pipette, we extracted the intermediate cloudy phase of the centrifuged protoplasts (~10 mL) from each tube while moving the syringe in circles. The remaining cells in each tube were then carefully pipetted into a 50 mL Falcon tube containing 5 mL W5, and brought to 10 mL by streaming W5 down the side of the tube. After returning deceleration to max (all following steps will be using max deceleration), the two tubes of cells were centrifuged at 100× *g* for 5 min at room temperature. We then carefully removed the supernatant without disturbing the pellet and added 10 mL of W5 to resuspend the protoplasts before centrifuging at 100× *g* for 2 min at room temperature. This process was repeated to further wash the cells. After the second resuspension, the number of cells per 100 µL was calculated using a hemocytometer (Number of protoplasts/mL = counted cells × 10^4^). The cells were again centrifuged at 100× *g* for 2 min, resuspended in MMG (4 mM MES, 0.4 M Mannitol, 15 mM MgCl_2_) buffer to adjust the concentration to 10^6^ cells/mL.

Individual 200 µL aliquots of the 10^6^ cells/mL MMG & protoplast solution were added to 1.5 mL microfuge tubes containing 40 µL of the plasmid DNA intended for transfection. These solutions were mixed with an equal volume (240 µL) of 40% PEG solution, the tube was gently flicked and then incubated for 20 min at room temperature. To stop the reaction, approximately 2x volume of W5 was added, and the tubes were mixed by inverting several times. The cells were centrifuged at 250× *g* for 5 min, and the supernatant was discarded by inverting the tube. Cells were washed once more with 800 µL W5. Pelleted cells were gently resuspended by pipetting into 1 mL of W5 with a cut tip and transferred into an additional 1 mL of W5 in a 6-well plate, where the cells were incubated in the dark for 48 h at room temperature. After the dark incubation, we performed reporter testing or DNA extractions using the cells. All experiments testing dual luciferase expression levels or gene editing were performed with at least three trial replicates of approximately 0.25–0.5 million cells from the total isolated cells used per treatment.

### 4.4. Improved PCR Efficiency with Genomic DNA Digestion

To isolate the genomic DNA for PCR, the protoplasts were first vortexed and then spun down at 12,000 rpm for 2 min in a centrifuge to burst the cells. The DNA present in this cell lysate can be used as the template for PCR amplification. To boost PCR efficiency, the lysate was treated with an off-target restriction enzyme, which targets a site not in the amplicon. This treatment shears up much of the background DNA, enabling more efficient PCR amplification compared to the untreated genomic DNA (Supplemental Figure S3A). PCR products were purified by gel band extraction from an agarose gel or by treatment with Exo-SapIT before being submitted for Sanger sequencing (Supplemental Figure S3B).

### 4.5. Agrobacterium Perfusion into Young Sunflower Leaves

Sunflower leaf infiltrations were performed similarly to the approach used in *N. benthamiana* transient assays [47], but with several key distinctions. *Agrobacterium* cultures were grown first over two nights in LB with antibiotics, split to an OD_600_ of 0.2, and subsequently treated overnight in AB:MES200 salt solution (17.2 mM K_2_HPO_4_, 8.3 mM NaH_2_PO_4_, 18.7 mM NH_4_Cl, 2 mM KCl, 1.25 mM MgSO_4_, 100 μM CaCl_2_, 10 μM FeSO_4_, 50 mM MES, 2% glucose (*w*/*v*), 200 μM acetosyringone, pH 5.5), akin to the Fast-TrACC method for seedling co-cultures [19]. To ensure we worked with leaves with higher potential for stomatal permeability, seedlings were sterilely grown in deli tubs in the same manner as used for the plants grown for protoplast isolations. Infiltrations were performed on the first true leaves, as they generally proved most amenable for delivering a greater volume of solution by perfusion. The infiltrated leaves were assessed for reporter signal after 7 days, allowing enough time for reporter gene expression in the transfected tissues.

### 4.6. CRISPR/Cas9 Reagent Gene Editing Efficiency Assessment

Using the CTAB protocol [57], DNA was extracted from sections of tissue positive for CmYLCV::AmCyan or CmYLCV::RUBY reporter signals (Supplemental Figures S4 and S5A,B). The AmCyan fluorescent portions of positive tissues were defined by using the NIGHTSEA Xite fluorescent flashlight and glasses system (Nightsea, Hatfield, PA, USA) and subsequently excised. After genomic DNA isolation, the targeted sunflower genes *FLOWERING LOCUS T-like 1* (*HaFT1* [58], HanXRQr2_Chr06g0274231) and *LYSOPHOSPHATIDIC ACID ACYLTRANSFERASE 3* (*HaLPAT3,* HanXRQr2_Chr01g0013171) were amplified with Phusion polymerase (New England Biolabs, Ipswich, MI, USA) and gene-specific primers (Figure 4B,C, Supplemental Table S2, Supplemental Figures S4 and S5). PCR amplicons were confirmed with gel electrophoresis, and if single bands of the correct size were observed, the PCR reaction was treated with Exo-SapIt (Thermo Fisher Scientific, Waltham, MA, USA) to prepare for Sanger sequencing on an 3730xl DNA Analyzer (Applied Biosystems, Waltham, MA, USA) by the UC Berkeley DNA Sequencing Facility (Berkeley, CA, USA).

Gene editing sequence analysis was performed using the Sanger sequences and modified peak assessment tools. To ensure that the results were not an artifact of a given software, both TIDE [59] and ICE [60] were employed to determine estimated levels of editing. Additionally, when submitting the samples for sequencing, multiple replicates from each tissue were submitted to ensure consistency in results (Supplemental Figure S5C–F).

### 4.7. Protoplast and Leaf Section Imaging

Protoplasts were visualized 48 hrs after transfection. Using the Z1 AxioObserver), protoplasts were initially assessed for cell viability at a zoom of 10X in bright field (Figure 1A). Transformed cell counts were made by combining the number of reporter-positive cells across 10 images taken at a higher 20X zoom, due to the signal being faint in some cells (Figure 1B,C).

To broadly detect reporter expression from infiltrated leaves, whole leaves were imaged to observe the extent of total delivery (Figure 2A–C). Photos of leaves treated with reagents to deliver a RUBY reporter were taken with a standard smartphone camera. Before luciferase imaging, leaves treated with reagents to deliver a luciferase reporter were submerged in water with 125 µM luciferin and shaken for 5 min while wrapped in foil. Imaging was performed with the IVIS in vivo imager (Revvity, Waltham, MA, USA) at an image scale covering a 7.5 cm × 7.5 cm scale. Whole leaves treated with reagents to deliver an AmCyan reporter were visualized with the RB-GO Xite Fluorescence Flashlight System (Nightsea, Hatfield, PA, USA).

For *Agrobacterium* delivery efficiency comparisons, we determined the intensity and area of AmCyan reporter transgene delivery by the candidate strains, EHA105 and AGL1, using the Axio Zoom V16 scanning microscope (Zeiss, Oberkochen, Germany). Images were taken at a consistent 7X zoom to ensure the same area was being assessed across the *Agrobacterium* treatments. Fluorescent images were quantified in ImageJ version 1.54g for AmCyan area and intensity analyses. From the AmCyan images, the fluorescent area was isolated by adjusting the threshold maximum on an image-specific basis, and the area and intensity were calculated using the “measure” and “analyze” functions.

### 4.8. Dual Luciferase Promoter Assessment

Dual luciferase assays were performed using the Promega Dual Luciferase^®^ Reporter Assay System (Promega, Madison, WI, USA; Cat. E1910) [61]. To determine the expression values for three well-established plant promoters (35S, CmYLCV, and AtUbi10), we used constructs driving Firefly luciferase expression from each of those promoters and driving expression of the control Renilla luciferase from the nos promoter (Supplemental Table S1) [56]. For protoplast dual luciferase experiments, 15 ug of construct DNA was transfected into 500,000 cells for each of the variable promoter constructs. After two days, the protoplasts were spun vigorously to burst the cells (12,000 rpm) and resuspended in passive lysis buffer before storing at −80 °C, where they may be stored for weeks.

For leaf infiltrations, *Agrobacterium* cultures carrying the same plasmids as used in the protoplast experiments were perfused into young sunflower leaves. One week after infiltration, tissue was collected on liquid nitrogen and ground with a mortar and pestle. This powder was resuspended in passive lysis buffer −80 °C, where it may be stored for weeks.

Before use in the dual luciferase assay, tissue samples in passive lysis buffer were vortexed to redistribute the plant tissue in the buffer. From each sample, 20 µL of the lysate is added to the wells of a black 96-well plate in triplicate. Firefly and Renilla substrate reagents were then prepared before adding 100 µL Firefly reagent to each well and subsequently reading luminescence on a Biotek Synergy 2 microplate reader (Agilent, Santa Clara, CA, USA). The Firefly reaction was then quenched with the Renilla reagent, and luminescence was again read on the plate reader. The average of the non-transfected control sample was used to establish the background on the machine, and each of the experimental Firefly luminescence values, related to the different tested promoters (35S, AtUBQ10, CmYLCV), was normalized to the Renilla luminescence values.

### 4.9. Agrobacterium and Cotyledon Co-Cultures

Sunflower seeds were shelled and sterilized as described above. After sterilization, the kernels were transferred to sterile 6-well plates (MedSupply Partners, Atlanta, GA, USA; GR-657185). In each well containing 2 mL of liquid ½ MS, three seeds were added and allowed to germinate. While the seeds were germinating, *Agrobacterium* was prepared following the Fast-TrACC protocol [19]. Three days before expected germination, cultures of the desired *Agrobacterium* strain were inoculated in LB with counter-selecting antibiotics and allowed to grow for two days to reach sufficient confluence. The day before co-culture, the *Agrobacterium* culture was spun down and resuspended in AB:MES200 at an OD600 concentration of approximately 0.2 and grown overnight. On the day of the co-culture, the *Agrobacterium* was spun down again and then resuspended in a 50:50 (*v*/*v*) mix of AB:MES200 salt solution and ½ MS liquid plant growth medium (1/2 MS salt supplemented with 0.5% sucrose (*w*/*v*), pH 5.5) at an OD600 of 0.15. This treated *Agrobacterium* solution was then added to cotyledons cut from the germinating seedlings. The cotyledon explants were co-cultivated with the *Agrobacterium* solution for two days before being washed with sterile ddH20 and transferred into a ½ MS salt and 0.5% sucrose (*w*/*v*) solution with 100 μM timentin. Reporter gene expression was assessed 5–7 days after removal from co-culture.

### 4.10. Statistical Analyses

ANOVA analyses were used to determine the significance values for the protoplast PEG transfection time experiment (Figure 1D) and for the Dual Luciferase promoter expression comparison (Figure 3D). To minimize variation in the Dual Luciferase expression quantification assay, each construct was tested with both three biological replicates and three technical replicates. Technical replicates were averaged to determine the mean expression result before statistical analysis. For the pairwise *Agrobacterium* comparisons between AGL1 and EHA105, *t*-tests were performed to determine statistical significance. This includes the comparisons between the fluorescence area (Figure 2H: 25 images/treatment) and fluorescence intensity (Figure 2I: 25 images/treatment). Unless otherwise stated, asterisks denote levels of significance corresponding to *p*-values < 0.05 (*), *p*-values < 0.01 (**), and *p*-values < 0.001 (***).

## 5. Conclusions

The focus of this study has been to establish a foundational toolkit for testing molecular reagents in sunflowers. The experiments conducted allowed for the determination of optimal input levels and PEG treatment times for protoplast transfection, identification of high-expression promoters for leaf infiltration, and comparison of *Agrobacterium* strain transfection rates. In some instances, tools for testing molecular functions were implemented that had not previously been demonstrated as effective in sunflower tissues, including the Dual Luciferase assay for comparative evaluation of promoter strength. Overall, our findings offer valuable new methods that can be directly employed to test components for functional experiments in sunflowers.

## Data Availability

All data generated or analyzed during this study are included in this published article. The newly assembled constructs in Supplementary Table S1, and their corresponding DNA sequences, are available on request to the authors.

## Supplementary Materials

The following supporting information can be downloaded at: https://www.mdpi.com/article/10.3390/plants15010089/s1, Figure S1: Parameters to Isolate and Transfect Healthy Sunflower Protoplasts; Figure S2: Impact of PEG Treatment Time on Transfection Rate and Cell Viability; Figure S3: Defining Effective PCR Conditions to Amplify Target Sequences from Protoplast DNA; Figure S4: Putative Gene Editing as Indicated by Sanger Traces Amplified from Transfected Protoplasts; Figure S5: Gene Editing Assessment in Leaf Infiltrated Tissues; Table S1: Constructs; Table S2: PCR Amplification and Sequencing Primers; Table S3: gRNA Sequences.
