# Supplementary material for: Establishing Reagent Testing Platforms for Functional Analyses in Sunflower
Source: Plants (Basel). 2025 Dec 27;15(1):89. doi: 10.3390/plants15010089 (PMC12788046; doi:10.3390/plants15010089)
Supplement: Supplementary file 1 [file plants-15-00089-s001.zip › plants-4010863-supplementary.pdf]

Establishing Reagent Testing Platforms for Functional Analyses in Sunflower

Supplemental Figures & Tables

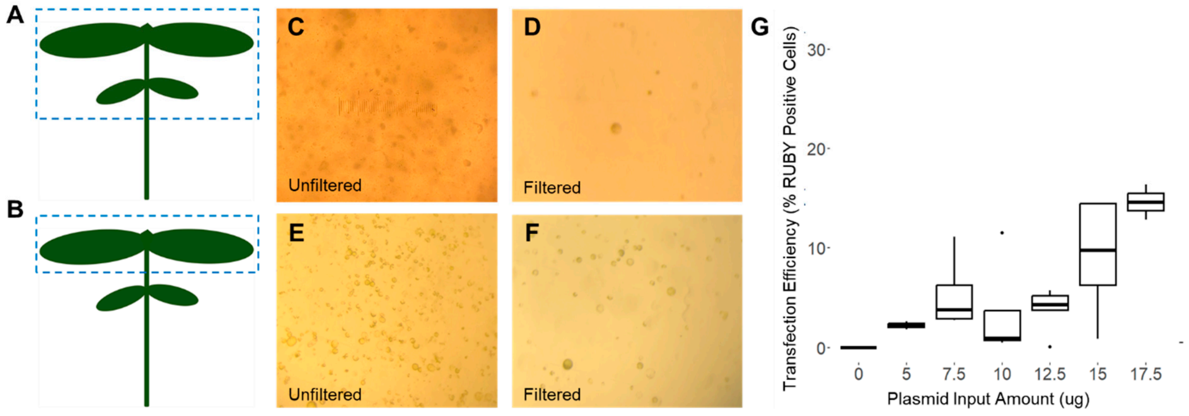

<u>Supplemental Figure S1. Parameters to Isolate and Transfect Healthy Sunflower Protoplasts</u>. Sunflower tissues including the cotyledons and true leaves (A, C, E) or only the true leaves (B, D, F) were selected for protoplast isolations. Even in the post-digest before filtering, much higher rates of debris were observed in isolations with cotyledons present (C) compared to isolations using only the true leaves (D). This debris in turn impacted the quantity and quality of filtered protoplasts between the cotyledon combined tissue treatment (E) and the true leaf only treatment (F). To further optimize the protoplast transfections, the amount of plasmid used for transfections was varied. Only experiments with plasmid inputs of 15ug or greater resulted in transfection rates consistently greater than 10% (G). All plasmid input concentration experiments were tested with 3 or more trials.

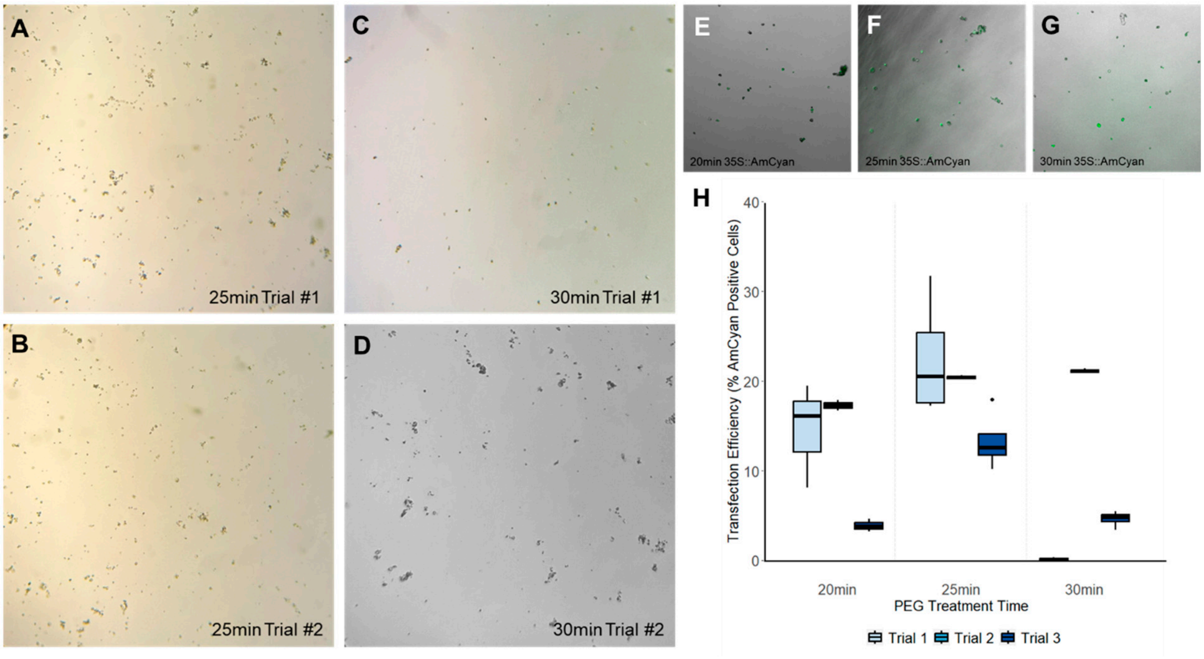

<u>Supplemental Figure S2. Impact of PEG Treatment Time on Transfection Rate and Cell Viability</u>. Looking to improve the protoplast transfection rate, the PEG treatment time was adjusted in 5-minute increments. While protoplast viability was consistently good up to the 25-minute PEG treatment (A-B), the 30-minute treatment showed considerable variability in the cell viability with some treatments yielding low levels of cells (C), seemingly due to the cells bursting, while others maintained high cell levels (D). When looking at the actual transfection rates, by assessing AmCyan reporter presence (E-G), the rates obtained with the 25-minute PEG treatment was most consistently high (H). While the 30-minute treatment showed a similarly high transfection rate in one trial (G-H), more trials failed due to cells bursting than succeeded.

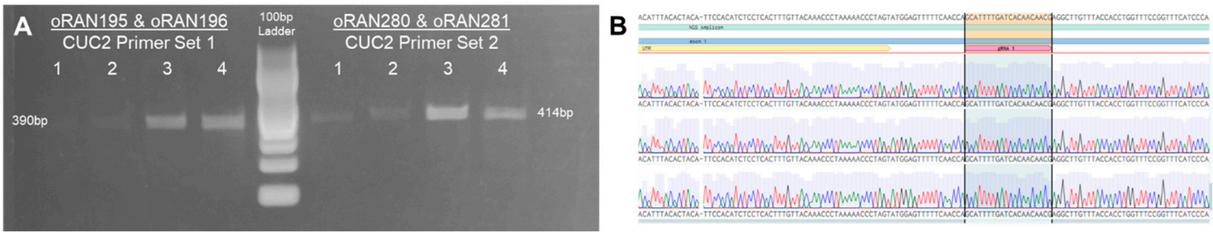

<u>Supplemental Figure S3. Defining Effective PCR Conditions to Amplify Target Sequences from Protoplast DNA.</u> DNA isolated from protoplast cells burst by centrifugation was used for PCR amplification and sequencing. In order to most effectively amplify the gene target, an off-target DNA digest was employed where a restriction enzyme not in the amplicon (BamHI, in this case) was used to pre-treat the genomic DNA. Comparing the non-digested (A, lanes 1 & 2) to the digested samples (A, lanes 3 & 4), the digestion treatment greatly improves amplification across PCR amplicons, which in turn can be submitted for Sanger sequencing (B).

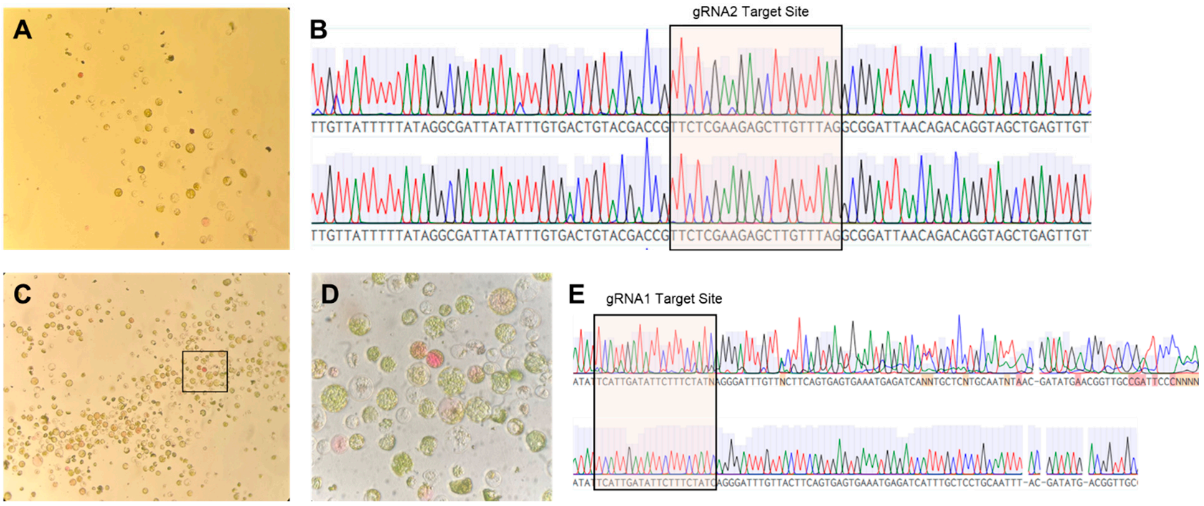

Supplemental Figure S4. Putative Gene Editing as Indicated by Sanger Traces Amplified from Transfected Protoplasts. Using the digest and amplification procedure, DNA from protoplasts treated with gene editing reagents targeting *HaLPAT3* was amplified for editing detection. DNA was isolated from protoplasts with positive RUBY reporter signal (A, C-D). Targets for two separate gRNAs (gRNA1 & gRNA2) were assessed with Sanger sequencing (B, E). No editing was observed across treatments for gRNA2 (B). Minimal trace variation was observed for gRNA1 treatments (E), suggesting low if observable editing.

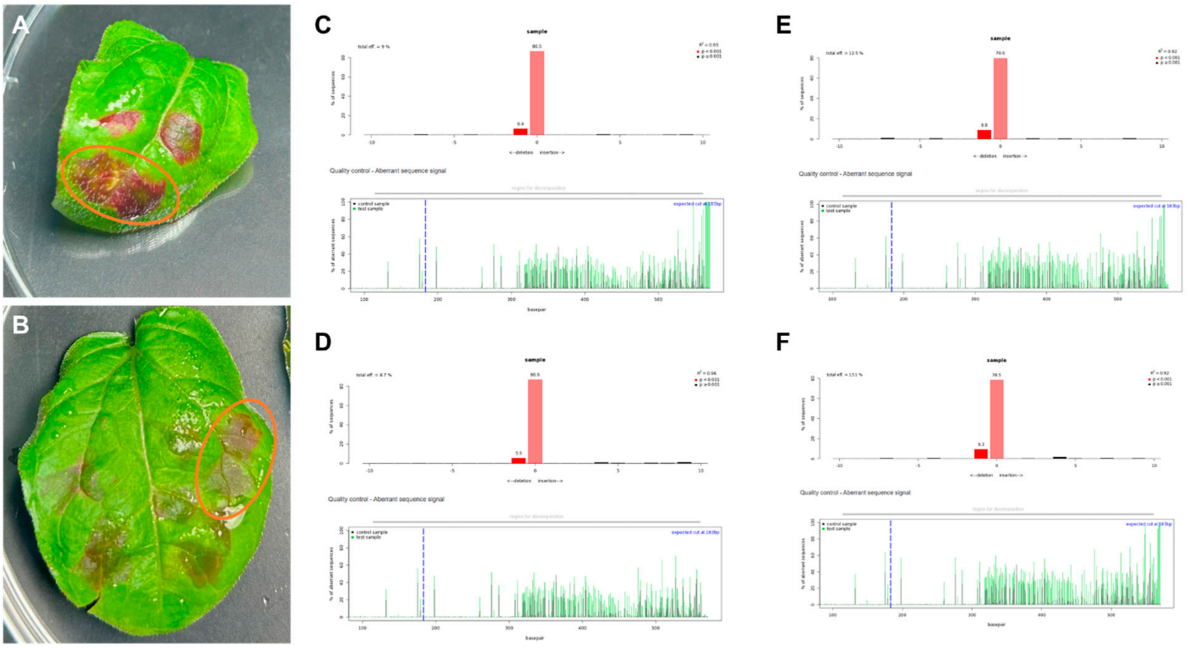

<u>Supplemental Figure S5. Gene Editing Assessment in Leaf Infiltrated Tissues</u>. To best determine the editing efficiency of different editing components, leaf infiltrated sites that presented the RUBY reporter signal (A-B, orange circle) were collected for DNA extraction. Tissue with either strong red pigmentation from a single (A) or multiple (B) perfusions were chosen. To confirm rates of editing, multiple PCR amplicons for different biological replicates (biological rep 1: A – amplicon 1, B – amplicon 2; biological rep 2: C – amplicon 1, D – amplicon 2) for a genomic target were assesses with the TIDE sequencing trace analyzer to determine rates of editing. For the different amplicons, the aberrant sequences (bottom, green bars) were downstream of the guide site as expected. Across amplicon technical replicates (A versus B; C versus D) the predicted editing tended to be consistent with more variation seen in the biological replicates (A & B versus C & D).

Plasmid Name	Content
pRAN73	CmYLCV::RUBY
pRAN210	35S::Luciferase, 35S::AmCyan
pAS6	AtU6::HaLPAT3 gRNA1
pAS8	AtU6::HaLPAT3 gRNA2
pAS10	35S::Cas9, AtU6::HaLPAT3 gRNA1, CmYLCV::RUBY
pAS12	35S::Cas9, AtU6::HaLPAT3 gRNA2, CmYLCV::RUBY
pRAN268	35S::Cas9, AtU6::FT gRNA1, CmYLCV::AmCyan, 35S::ipt
pRAN269	35S::Cas9, AtU6::FT gRNA2, CmYLCV::AmCyan, 35S::ipt
External Plasmids	
A0101{cite}	35S::Cas9
pJC616{cite}	35S::FLuc, nos::Renilla
pJC618{cite}	CmYLCV::FLuc, nos::Renilla
pJC619{cite}	AtUbi10::FLuc, nos::Renilla

Supplemental Table S1. Constructs

Primer Name	Primer Sequence	Primer Use
oRAN97	CAAATCTCACTACCGATTTGCAC	HaLPAT3 Sequencing Fwd
oRAN98	TATCCGCTTATTCCAGAGTG	HaLPAT3 Sequencing Rev
oRAN129	CCAACAGACTTGAAAGAAACAAC	HaFT Fwd Sequencing Primer
oRAN130	CGTAGCTTAAGCTTTTTGTTCTTT	HaFT Rev Sequencing Primer
oRAN195	CCTCACTTTGTTACAAACCC	HaCUC2 Fwd Sequencing Primer
oRAN196	CGTAGACTAAAGAAGTACCAC	HaCUC2 Rev Sequencing Primer
oRAN280	CTTGATTCTGTGAGAACACC	HaCUC2 Fwd Sequencing Primer
oRAN281	CAAAACCCTAAAAACATTATGTG	HaCUC2 Rev Sequencing Primer

Supplemental Table S2. PCR Amplification & Sequencing Primers

**Target Gene**	**gRNA Number**	**Spacer Sequence**
HaFT	gRNA1	GAGGAGGGAGAGGGATTCGT
HaFT	gRNA2	CTCAGGTTATTAACCAGCCT
HaLPAT3	gRNA1	TCATTGATATTCTTTCTATC
HaLPAT3	gRNA2	CTAAACAAGCTCTTCGAGAA

Supplemental Table S3. gRNA Sequences
